# Urate-lowering therapy in cardiovascular pharmacotherapy: from Mendelian randomization data to cardio–renal–metabolic risk modulation

**DOI:** 10.1093/ehjcvp/pvag042

**Published:** 2026-06-17

**Authors:** Claudio Borghi, Federica Fogacci, Arrigo F G Cicero

**Affiliations:** Hypertension and Cardiovascular Risk Research Center, Medical and Surgical Sciences Department, Alma Mater Studiorum University of Bologna, Via Massarenti 9, Bologna 40138, Italy; Hypertension and Cardiovascular Risk Research Center, Medical and Surgical Sciences Department, Alma Mater Studiorum University of Bologna, Via Massarenti 9, Bologna 40138, Italy; Department of Medical Pharmacology, Medical Faculty, Ataturk University, Erzurum 25240, Turkey; Hypertension and Cardiovascular Risk Research Center, Medical and Surgical Sciences Department, Alma Mater Studiorum University of Bologna, Via Massarenti 9, Bologna 40138, Italy; Cardiovascular Medicine Unit, Heart, Chest and Vascular Department, IRCCS Azienda Ospedaliero-Universitaria di Bologna, Via Massarenti 9, Bologna 40138, Italy

**Keywords:** Urate-lowering therapy, Cardiovascular disease, Serum uric acid, Mendelian randomization

## Abstract

**Aims:**

Serum uric acid (SUA) and hyperuricaemia have re-emerged as determinants of cardiovascular (CV) risk beyond gout. This review integrates epidemiological, Mendelian randomization (MR), and pharmacological evidence to define the role of SUA and urate-lowering therapy (ULT) in contemporary CV pharmacotherapy.

**Methods and results:**

We synthesized population-based studies, MR and drug-target MR analyses, and randomized trials of xanthine oxidase inhibitors (XOIs), uricosurics/URAT1 inhibitors, biologic uricases, and CV drugs with urate-modifying effects. Hyperuricaemia is prevalent and rising, often closely accompanying obesity, hypertension, diabetes, and chronic kidney disease (CKD). Higher SUA correlates with coronary artery disease, heart failure, stroke, cardio–renal–metabolic syndromes, and mortality, with non-linear risk relationships and sex- and age-specific thresholds. Mendelian randomization suggests a modest causal contribution of genetically elevated SUA to blood pressure, coronary disease, and advanced CKD, partly mediated via haemodynamic, renal, and inflammatory pathways. Pharmacologically, XOIs, URAT1 inhibitors, and uricases differ in kinetics and safety profiles. However, large trials and real-world cohorts show no consistent reduction in CV events when ULT is added to guideline-directed therapy in asymptomatic hyperuricaemia, stable ischaemic heart disease, chronic heart failure, or CKD. Observational data suggest that long-term, adequately dosed XOIs and high cumulative uricosuric exposure may reduce coronary risk.

**Conclusion:**

Serum uric acid is a cardio–renal–metabolic biomarker and a plausible therapeutic target in selected cardio–renal–metabolic phenotypes. However, evidence does not support routine ULT for CV prevention in asymptomatic hyperuricaemia. Urate-lowering therapy should remain focused on gout and symptomatic hyperuricaemia, with phenotype-guided strategies to identify patients with asymptomatic hyperuricemia most likely to benefit.

## Graphical Abstract

**Graphical Abstract:**
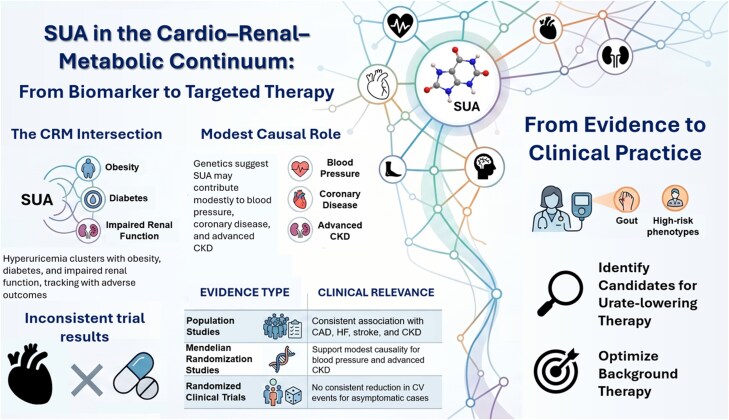

## Introduction

Cardiovascular disease (CVD) remains the leading global cause of morbidity and mortality despite substantial advances in lipid lowering, blood pressure control, and glucose management.^1^ Here, residual cardiovascular risk refers to the risk that persists despite guideline-directed management of established modifiable risk factors.^2^ Against this residual risk, serum uric acid (SUA) and hyperuricaemia have re-emerged as potential contributors to the cardio–renal–metabolic (CRM) continuum beyond gout.^3,4^ Hyperuricaemia is common and increasing across populations,^5–7^ and elevated SUA consistently associates with hypertension, coronary artery disease (CAD), heart failure (HF), stroke, chronic kidney disease (CKD), and mortality.^8–12^

Serum uric acid is biologically pleiotropic: the ancestral loss of uricase may have been adaptive in low-salt, low-calorie environments,^13^ whereas in modern settings it may foster hypertension, insulin resistance, and vascular injury.^3,13^ Mechanistic data link SUA to oxidative stress, endothelial dysfunction, vascular remodelling, renin–angiotensin system activation, and inflammation [including NOD-like receptor family pyrin domain containing 3 (NLRP3) signalling],^3,4,14^ while transporter-mediated handling across kidney and gut connects SUA to genetics, adiposity, ageing, and the microbiome.^3,15–17^

Causality, however, remains debated. Observational evidence ranges from early attenuation after multivariable adjustment in Framingham^8^ to more recent signals for hypertension and adverse cardiovascular outcomes, including non-linear mortality associations and stronger prognostic value in selected secondary-prevention phenotypes.^10,12,18,19^ Human genetics strengthens causal inference: genome-wide association studies (GWAS) implicate urate transporter loci,^3^ and Mendelian randomization (MR) supports a modest directional effect on blood pressure^20^ and links to CAD/MI and broader CKM phenotypes across populations.^10,21–23^ Yet randomized trials of urate-lowering agents show mixed results for hard CV endpoints, arguing against universal cardioprotection.

Accordingly, this review (i) summarizes the epidemiologic burden and cardio–renal–metabolic associations of hyperuricaemia, (ii) examines causal inferences from MR and drug-target MR, (iii) discusses the pharmacology of cardio-relevant urate-lowering agents, and (iv) critically appraises how these therapies relate to cardiovascular outcomes to clarify where urate-lowering therapy (ULT) fits within modern cardiovascular pharmacotherapy.

## Methods

This article was conceived as a narrative review providing a clinically oriented synthesis of evidence on SUA and ULT in cardiovascular pharmacotherapy. Relevant literature was identified through searches of major electronic databases, including PubMed/MEDLINE and Embase, with emphasis on epidemiological studies, MR analyses, pharmacological investigations, and randomized clinical trials. Priority was given to recent and clinically relevant publications. Additional references were retrieved from the bibliographies of key articles. As this was a narrative review, no formal systematic review methodology or PRISMA-based selection process was applied.

## Serum urate in the cardio–renal–metabolic continuum

### Prevalence, temporal trends, and clustering

Hyperuricaemia is now common in routine practice. In a cross-sectional analysis of >730 000 adults undergoing health examinations in Wuhan (China), prevalence reached 25.8% in 2019, rising to 36.6% in men and 10.8% in women.^5^ Over the preceding decade, median SUA increased in both sexes and age-specific prevalence rose across all strata, with the steepest relative increase among adults aged 20–39 years.^5^

Comparable secular trends are reported in Western cohorts. In Germany (1985–2005), mean SUA was 314.8 μmol/L in men and 243.6 μmol/L in women, increasing by ∼6.7 μmol/L per decade in men and rising sharply in women after 50 years; using sex-specific thresholds, hyperuricaemia exceeded 15% in men and 13% in women and became more prevalent in women aged >65 years.^6^ In Ireland, prevalence increased between 2006 and 2014 from 19.7% to 25.0% in men and from 20.5% to 24.1% in women, alongside higher mean SUA.^7^

Across cohorts, hyperuricaemia clusters with obesity/central adiposity, hypertension, diabetes, dyslipidaemia, and impaired renal function.^3,5,7,10^ In the China Health and Retirement Longitudinal Study (CHARLS), a nationally representative Chinese population-based cohort, and the US National Health and Nutrition Examination Survey (NHANES), a US-based population survey, SUA, asymptomatic hyperuricaemia, and gout tracked with CRM/cardio–kidney–metabolic (CKM) burden; among those with at least one condition, higher SUA and poorly controlled hyperuricaemia predicted higher all-cause mortality, whereas gout with normalized SUA did not.^10^ Body-shape trajectory analyses and MR further support adiposity as a causal driver, with genetically predicted childhood and adult body mass index (BMI) increasing SUA.^15^ Overall, hyperuricaemia emerges as a prevalent and growing exposure embedded within the broader CRM/CKM milieu, rather than a niche abnormality confined to gout or advanced CKD.

### Cardiovascular and renal outcomes, thresholds, and non-linear associations

Beyond risk-factor clustering, hyperuricaemia has been linked to a broad range of cardiovascular and renal outcomes. Prospective cohorts report higher rates of CAD, stroke, HF, and CKD in individuals with elevated SUA even after adjustment for traditional risk factors^8,10,11^; in a cohort of >4000 participants, elevated SUA, or xanthine oxidase inhibitor (XOI) use (as a proxy for hyperuricaemia) tracked with higher incidences of these outcomes and a greater burden of hypertension, obesity, and diabetes.^11^

In individuals with established CVD, analyses from the US National Health and Nutrition Examination Survey (NHANES) showed that higher SUA was associated with increased all-cause and CV mortality in a non-linear pattern, with spline models demonstrating an L-shaped association and a sharp rise in risk beyond ∼6.0–6.1 mg/dL.^9^ Consistently, a meta-analysis of >1 million individuals linked hyperuricaemia to higher risks of incident CHD, CHD death, CVD death, and myocardial infarction, with approximately linear risk increases across SUA levels and a U-shaped SUA–CVD mortality relationship in men.^12^ Serum uric acid may also refine residual risk in treated CAD: in a large secondary-prevention cohort, higher SUA predicted more major adverse cardiovascular and cerebrovascular events (MACCEs), particularly in patients with 0–1 standard modifiable cardiovascular risk factors (SMuRFs).^19^

Several cohorts suggest sex-specific thresholds, generally lower in women. In URRAH, SUA ≥5.6 mg/dL predicted cardiovascular mortality, while all-cause mortality thresholds were 5.4 mg/dL in men and 4.7 mg/dL in women^24^; other European^25^ and Japanese cohorts^26^ similarly report excess risk at ≥7.0 mg/dL in men but ∼5.0–6.0 mg/dL in women. Overall, these data support SUA as a continuous, context-dependent risk marker, with non-linear associations shaped by sex, age, and comorbidity burden. Women may reach clinically relevant risk at lower SUA levels than men, and the age-related increase in SUA appears particularly pronounced after menopause, supporting a sex-informed interpretation of SUA in cardiovascular and cardio–renal–metabolic risk assessment.^6,24–26^ However, current evidence does not yet justify distinct sex-specific urate-lowering therapy targets for cardiovascular prevention.

## From association to causality: human genetics and drug-target Mendelian randomization

Serum urate is highly heritable, and GWAS have identified multiple SUA-regulating loci, including renal/intestinal transporters and purine-metabolism enzymes.^3^ This is pharmacologically relevant because several determinants of SUA constitute drug targets (e.g. xanthine oxidase and URAT1).

Mendelian randomization supports a modest causal role for SUA. In European two-sample MR, genetically higher SUA increased systolic (β = 0.136, 95% CI 0.035–0.238) and diastolic blood pressure (β = 0.108, 95% CI 0.007–0.209), with no convincing reverse effect, consistent with observational links to incident hypertension.^8,20^ Mendelian randomization also implicates SUA in CAD/MI: in East Asians, SUA-raising variants increased CAD risk and were associated with adverse cardio–renal–metabolic traits^21^; cross-trait MR linked SUA to CAD, stable angina and MI, partly mediated through blood pressure and triglycerides.^22^ Within a CKM framework, genetically higher SUA was associated with CVD, CKD, diabetes, and advanced CKM stages independent of renal function.^10,23^

Drug-target MR further prioritizes pathways: proxies for URAT1-related transporters (SLC22A11/SLC22A12; lesinurad-like inhibition) were associated with lower ischaemic heart disease risk,^23^ while SLC2A9 (GLUT9) variants link SUA with eGFR^27^ and may confer a more favourable cardiometabolic profile than URAT1 inhibition in certain analyses.^28^ Overall, genetics suggests SUA is more than a biomarker but a modest causal contributor to blood pressure and atherosclerotic/CKM phenotypes^10,20–23^; however, MR reflects lifelong exposure and may not predict the effect size of shorter-term pharmacologic lowering.

## Urate-lowering drugs in cardiovascular pharmacology

The urate-lowering drugs in current use are mainly the XOIs, followed by the uricosurics and transporter inhibitors, and only rarely the biologic uricases.

### Xanthine oxidase inhibitors

Xanthine oxidase inhibitors, mainly allopurinol and febuxostat, lower SUA by inhibiting xanthine oxidase-mediated urate production and remain the mainstay of chronic ULT in gout. A recent review concludes that long-term XOI therapy is effective, safe, and generally well tolerated, including in older adults and in patients with HF or cancer.^29^ Beyond urate lowering, XOIs may also ameliorate oxidative stress, endothelial function, and renal haemodynamics.^29–32^

In a model-based meta-analysis of 49 trials (10 591 participants), XOIs reduced SUA by ∼35% at 3 months, with an efficacy plateau after ∼7 weeks; estimated glomerular filtration rate (eGFR) increased modestly (+0.7%) after 1 year, consistent with a small renoprotective effect.^33^ Although ∼75% of trial participants on urate-lowering drugs achieved SUA <6 mg/dL, real-world target attainment is considerably lower.^32,33^ Safety data from the same meta-analysis showed adverse events in ∼56%, serious events in 4% and discontinuation in 17%.^33^ Allopurinol has the longest safety record, but rare severe hypersensitivity requires vigilance and appropriate dosing, particularly in CKD.^34^ Febuxostat is generally well tolerated, though cardiovascular safety has remained debated (see below).

Overall, XOIs remain first-line ULT for most patients, including many with concomitant CVD or CKD, and therefore stand at the centre of any cardiovascular pharmacotherapy strategy that incorporates urate lowering.^29–34^

### Uricosurics and transporter inhibitors

Uricosurics (e.g. probenecid and benzbromarone) increase renal urate excretion by inhibiting tubular reabsorption, mainly via URAT1, and can be used as monotherapy in patients with preserved renal function or as add-ons to XOIs. In meta-analytic data, URAT1 inhibitors reduced SUA by ∼37.5% at 3 months, marginally more than XOIs, but were associated with a small eGFR decline at 1 year (−2.5%).^33^ Adverse events occurred in ∼52%, serious events in 2.4%, with an 8% discontinuation rate.^33^ Key limitations include hepatotoxicity issues with benzbromarone and drug–drug interactions with probenecid.^34^

More selective URAT1 inhibitors (lesinurad and verinurad) permit potent urate lowering, often in combination with XOIs; although clinical experience is presently limited, safety appears acceptable when used appropriately.^32,34^ Drug-target MR implicating SLC22A11/SLC22A12 offers a genetic rationale to explore URAT1 inhibition in high-risk cardio–renal–metabolic phenotypes and ischaemic heart disease (IHD).^23^ GLUT9 (SLC2A9) is another candidate: MR suggests SLC2A9 modulation influences both SUA and kidney function, raising the prospect of urate lowering with concomitant renal benefit.^27^

### Biologic uricases

Biologic uricases (rasburicase and pegloticase) provide rapid and profound urate depletion by converting uric acid into allantoin. They remain reserved for tumour lysis syndrome and severe, refractory gout. In the meta-analysis, uricase-based therapies reduced SUA by nearly 80% at 3 months, but at the expense of high rates of gout flares (over 50% in the first 3 months), adverse events (over 90%), serious events (nearly 19%), and treatment discontinuation (∼31%).^33^ Immunogenicity and infusion reactions remain major limitations.^32,34^

From a cardiovascular perspective, uricases fill a narrow niche: they may be crucial for patients with severe tophaceous gout and high cardio–renal–metabolic burden, where rapid urate debulking could reduce systemic inflammation and functional limitation, but they are not suitable for routine cardiovascular prevention. Class-level differences in urate-lowering efficacy, renal effects, and safety across XOIs, URAT1 inhibitors and biologic uricases are outlined in *Table 1*.

**Table 1:** Summary of key pharmacological characteristics of XOIs, uricosurics/URAT1 inhibitors and biologic uricases, including expected SUA lowering, renal effects, safety, and practical renal and cardiovascular considerations

Drug class and representative agents	Mechanism and urate-lowering effect	Renal and safety profile	Renal and cardiovascular considerations
XOIs: allopurinol, febuxostat, topiroxostat	Inhibit xanthine oxidase and reduce urate production. In meta-analytic data, XOIs lower serum urate by ∼35% at 3 months, with an efficacy plateau around week 7.^33^	Associated with a small increase in eGFR at 1 year, ∼+0.7%^33^. Adverse events occur in about 56% of patients, serious adverse events in about 4%, and treatment discontinuation in around 17%^33^.	XOIs remain the standard first-line option for most patients with gout and symptomatic hyperuricaemia. Their long clinical use supports an overall reassuring cardiovascular safety profile.^29,34^ Potential vascular or renal benefits have been proposed in selected phenotypes, although large trials in ischaemic heart disease, including ALL-HEART, did not show a reduction in hard cardiovascular outcomes.^30,31,44^
Uricosurics/URAT1 inhibitors: probenecid, benzbromarone, lesinurad, verinurad	Increase urinary urate excretion by inhibiting renal tubular urate reabsorption, mainly through URAT1 and related transporters. Serum urate reduction is ∼37–38% at 3 months, with a plateau around Week 10.^33^	Associated with a modest eGFR decline at 1 year, ∼−2.5% in meta-analysis.^33^ Adverse events occur in about 52% of patients, serious adverse events in about 2–3%, and discontinuation in around 8%^33^.	These agents are useful as add-on therapy to XOIs in difficult-to-control gout.^32^ They require caution in CKD and in patients with previous urolithiasis.^34^ Drug-target Mendelian randomization suggests that URAT1-related pathways may be relevant to ischaemic heart disease and advanced cardio-kidney-metabolic phenotypes, although cardiometabolic trade-offs may differ across urate transport pathways.^23,27,28^
Biologic uricases: rasburicase, pegloticase	Convert urate into allantoin, producing rapid and profound urate depletion. Serum urate reduction is ∼80% at 3 months in model-based meta-analysis.^33^	Often used in patients with advanced CKD, although systematic eGFR data are limited. Adverse events occur in more than 90% of patients, serious adverse events in about 19%, and treatment discontinuation in around 31% at 1 year.^33^	Biologic uricases are reserved for tumour lysis syndrome and severe refractory tophaceous gout.^32^ Because infusion reactions, immunogenicity, and discontinuation are frequent, their use is generally limited to selected patients, often with substantial cardiovascular or renal comorbidity.^34^ Cardiovascular benefit has not been established; treatment is aimed primarily at rapid control of severe urate burden.

CKD, chronic kidney disease; CV, cardiovascular; eGFR, estimated glomerular filtration rate; SUA, serum uric acid; URAT1, urate transporter 1; XOI, xanthine oxidase inhibitor.

### Cardiovascular and metabolic drugs that affect urate

Several cardiovascular and metabolic drug classes affect SUA as a secondary effect. SGLT2 inhibitors, losartan, some calcium channel blockers, ACE inhibitors, statins, and fenofibrate are generally either urate-neutral or urate-lowering.^32,36,37^ Among agents acting on the renin–angiotensin system, however, losartan appears unique because of its uricosuric effect.^37^ This effect is thought to be mediated by inhibition of URAT1-dependent tubular urate reabsorption; in a *post hoc* analysis of HEAAL in symptomatic HF, high-dose losartan reduced SUA by ∼0.27 mg/dL vs. low-dose therapy and reduced incident hyperuricaemia, although incident gout was unchanged.^37^ In contrast, thiazide and loop diuretics, many β-blockers, low-dose aspirin, and calcineurin inhibitors increase SUA and may trigger gout.^36^

**Figure 1:**
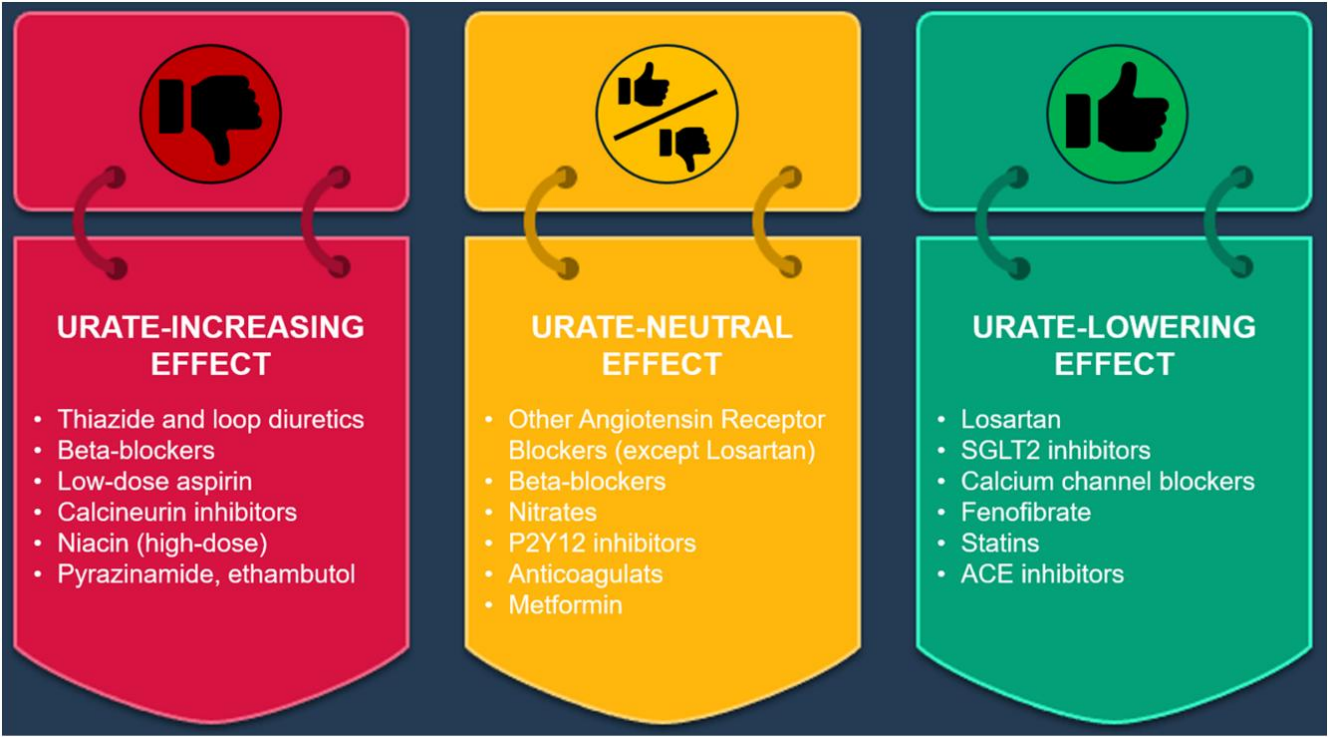
Effects of commonly used cardiovascular and metabolic drug classes on serum uric acid (SUA). Selected drug classes commonly used in cardiovascular practice are classified according to whether they predominantly lower, do not meaningfully modify, or increase SUA.

Sodium–glucose cotransporter 2 (SGLT2) inhibitors are especially attractive in hyperuricaemia patients with type 2 diabetes, CKD, or HF. In addition to their established cardioprotective and nephroprotective effects in large randomized trials,^32^ comparative effectiveness data indicate that, in metformin-treated patients with Type 2 diabetes, SGLT2 inhibitors are associated with lower risks of incident gout and recurrent flares, together with lower risks of major adverse cardiovascular events and HF, compared with sulfonylureas.^35^ Recent target trial emulation data further suggest that, in patients with gout and Type 2 diabetes, initiation of an SGLT2 inhibitor is associated with reduced subsequent allopurinol initiation, lower use of acute gout therapies and diuretics, and fewer recurrent gout flares, supporting a possible medication-sparing role in this high-risk population.^38^ Although in some cohorts empagliflozin did not significantly change SUA, it improved weight, blood pressure, lipids, and glycaemic control without worsening renal function in patients with T2D and established CAD^39^ and has robust cardioprotective and nephroprotective effects in large RCTs.^32^ Fenofibrate can lower both triglycerides and SUA and may be useful in patients with combined hypertriglyceridaemia and hyperuricaemia.^36^ A schematic summary of the effects of commonly used cardiovascular and metabolic drug classes on SUA is provided in *Figure 1*.

For cardiologists, these data underscore that part of ‘treating urate’ entails rational curation of background therapy: wherever feasible, favouring drugs that reduce CV risk and are urate-neutral or urate-lowering, including SGLT2 inhibitors and, where clinically appropriate, losartan, and minimizing unnecessary exposure to strongly urate-raising agents in patients with gout or high urate burden. In selected patients with gout and Type 2 diabetes, this may also include choosing therapies that help lessen reliance on conventional urate-lowering, flare, and diuretic treatments.^38^ The effects of commonly used cardiovascular and metabolic drug classes on SUA are summarized in *Table 2*.

**Table 2:** Overview of commonly used cardiovascular and metabolic drug classes that lower, are neutral to, or raise SUA, with practical implications for patients with hyperuricaemia or gout

Drug class	Effect on serum urate	Cardiorenal context	Practical considerations
SGLT2 inhibitors: empagliflozin, dapagliflozin, etc.^35,36^	Usually lower SUA modestly, largely through a mild uricosuric effect.	Provide established cardiovascular and renal protection in patients with type 2 diabetes, heart failure, and CKD.	Particularly suitable when indicated in patients with hyperuricaemia, gout, diabetes, CKD, or heart failure, as they combine cardiorenal benefit with a generally favourable urate profile.
Angiotensin II receptor blockers: especially losartan^34,37^	Losartan lowers SUA modestly through uricosuric activity; this effect is less consistent with other ARBs.	ARBs reduce blood pressure and provide cardiovascular and renal protection in hypertension and CKD.	Losartan may be preferred when an ARB is clinically appropriate in patients with hyperuricaemia or gout.
ACE inhibitors^36^	Generally neutral, although mild SUA reductions have been reported with some agents.	Core therapies for hypertension, heart failure, and CKD with albuminuria.	Reasonable background therapy in hyperuricaemic patients requiring RAAS blockade, with no major adverse urate signal.
Calcium channel blockers^36^	Neutral or mildly urate-lowering in several studies.	Effective antihypertensive agents with established cardiovascular benefit.	Useful alternatives to urate-raising antihypertensive drugs in patients with gout or elevated SUA.
β-Blockers^36^	Often associated with higher SUA.	Remain essential after myocardial infarction and in HFrEF, despite variable metabolic effects.	Their use should be maintained when clinically indicated, while accounting for their potential to increase SUA in patients with difficult-to-control gout.
Thiazide and loop diuretics^36^	Increase SUA, sometimes substantially.	Important for blood pressure control and volume management, particularly in heart failure and resistant hypertension.	Use the lowest effective dose where possible in patients with gout. When diuretics are unavoidable, monitor SUA, and manage gout risk proactively.
Statins^36^	Mostly neutral; some agents, such as atorvastatin, may slightly reduce SUA.	Cornerstone therapy for ASCVD prevention and treatment.	Concerns about SUA should not limit their use, as their cardiovascular benefits far outweigh any minimal or neutral effects on serum urate.
Bempedoic acid^58,59^	Increases SUA by ∼0.7–0.8 mg/dL on average and increases gout risk.	Lowers LDL-C and reduces cardiovascular events in statin-intolerant, high-risk patients.	Useful in selected statin-intolerant patients, but baseline SUA and gout history should be assessed. Closer monitoring is advisable in patients with hyperuricaemia or previous gout.
Fenofibrate^36^	Lowers SUA to a clinically relevant extent.	Lowers triglycerides and may improve atherogenic dyslipidaemia.	Particularly attractive in patients with hypertriglyceridaemia and coexisting gout or hyperuricaemia.
Low-dose aspirin^36^	Modestly increases SUA.	Essential for secondary prevention in patients with ASCVD.	Aspirin should generally be continued when clinically indicated; if gout occurs, it should usually be managed with dedicated urate-lowering therapy rather than aspirin withdrawal.
Calcineurin inhibitors: cyclosporine, tacrolimus^36^	Frequently increase SUA, sometimes markedly.	Used mainly in transplant medicine and selected autoimmune conditions; may also impair renal function.	SUA should be monitored closely. Early gout prevention or urate-lowering strategies may be needed in high-risk patients.

ACE, angiotensin-converting enzyme; ARB, angiotensin II receptor blocker; ASCVD, atherosclerotic cardiovascular disease; CKD, chronic kidney disease; CV, cardiovascular; HF, heart failure; HFrEF, heart failure with reduced ejection fraction; LDL-C, low-density lipoprotein cholesterol; RAAS, renin–angiotensin–aldosterone system; SUA, serum uric acid; T2D, type 2 diabetes.

## Urate-lowering therapy and cardiovascular outcomes

### Ischaemic heart disease

Observational studies suggested that allopurinol may improve cardiovascular outcomes through XO inhibition, with beneficial effects on endothelial function and oxidative stress.^30,31^ In a regional Swedish cohort of 18 862 patients with gout but no prior CHD, long-term allopurinol use was associated with lower odds of first-ever acute coronary syndrome (ACS), with adjusted ORs of 0.77 (95% CI 0.63–0.94) for 100 mg and 0.61 (95% CI 0.47–0.81) for >100 mg vs. non-use.^42^ High-cumulative exposure to allopurinol or benzbromarone was likewise associated with lower CAD risk in a Taiwanese gout cohort, whereas lower cumulative doses were not.^43^

By contrast, the ALL-HEART trial provided a definitive randomized evaluation of allopurinol in stable ischaemic heart disease without gout. In 5,721 patients aged ≥60 years with chronic IHD, high-dose allopurinol (up to 600 mg/day) added to usual care did not reduce the composite of non-fatal MI, non-fatal stroke, or cardiovascular death over a mean 4.8 years (HR 1.04, 95% CI 0.89–1.21) and did not alter all-cause mortality (HR 1.02, 95% CI 0.87–1.20).^44,45^

These findings highlight a central theme: ULT may be beneficial in selected gout populations with high urate burden and inflammation but does not improve outcomes when ‘added onto’ otherwise well-treated IHD in patients without gout.

### Heart failure, chronic kidney disease, and asymptomatic hyperuricaemia

Despite strong prognostic associations between SUA and outcomes in HF and CKD, trials targeting asymptomatic hyperuricaemia have remained neutral. In a multicentre randomized controlled trial of 101 Japanese outpatients with HF with reduced ejection fraction (HFrEF) and asymptomatic hyperuricaemia, febuxostat did not significantly change B-type natriuretic peptide (BNP) at 24 weeks nor improve left ventricular (LV) function, New York Heart Association (NYHA) class, renal function, or major CV events compared with control.^46^ In a retrospective cohort of 979 HF patients with asymptomatic hyperuricaemia, febuxostat lowered SUA but did not lower a composite of CV death and rehospitalization,^47^ although a higher SUA/creatinine ratio marked higher risk.

In CKD, two large Japanese new-user cohorts found no material difference in composite CV events or mortality between febuxostat and allopurinol,^48,49^ and no difference in time to kidney replacement therapy.^49^

At the population level, a large Japanese claims-based cohort of over 150 000 adults with asymptomatic hyperuricaemia (SUA ≥7.0 mg/dL) showed that ULT did not reduce incident CAD, stroke or atrial fibrillation compared with no therapy (HR 1.01, 95% CI 0.89–1.13).^50^

These data together argue against routine ULT as a cardiovascular preventive measure in asymptomatic hyperuricaemia.

### Comparative cardiovascular safety: allopurinol, febuxostat, and benzbromarone

The cardiovascular safety of febuxostat vs. allopurinol has prompted concern. An Austrian population-based cohort including 28 068 patients initiating febuxostat or allopurinol reported greater rates of non-fatal CV events and all-cause mortality in febuxostat initiators; the adjusted HR for the composite endpoint comparing allopurinol with febuxostat was 0.58 (95% CI 0.53–0.63).^51^

Conversely, the FAST trial randomized 6128 older gout patients, already optimized on allopurinol, to continue allopurinol or switch to febuxostat. Over a median 3.6 years of on-treatment follow-up, febuxostat was non-inferior to allopurinol for the composite CV endpoint (adjusted HR 0.85, 95% CI 0.70–1.03) and was not associated with excess mortality.^52^ In Korean gout patients, CV risk did not differ between allopurinol and benzbromarone initiators, even in high-risk subgroups.^53^

Overall, allopurinol appears safe from a cardiovascular standpoint and possibly beneficial in some contexts, while febuxostat appears acceptable in well-selected, closely monitored patients, although some real-world data suggest higher CV risk in unselected populations.^51,52^

### Periprocedural and mechanistic trials

In peri-interventional settings, ULT may confer short-term renal benefits. In hyperuricaemia CHD patients undergoing percutaneous coronary intervention (PCI), febuxostat plus hydration reduced the incidence of contrast-induced nephropathy from 14.7% to 6.0% and attenuated the rise in creatinine and neutrophil gelatinase-associated lipocalin compared with hydration alone.^54^

Mechanistic HFpEF data are less compelling. In a Phase 2 trial of 159 patients with HF with preserved ejection fraction (HFpEF) and elevated SUA, verinurad plus allopurinol produced ∼60% SUA reduction, vs. ∼38% with allopurinol and negligible change with placebo, but failed to improve peak oxygen consumption (peak VO2) or symptoms, and CV events were similar across arms.^40^ After recent ischaemic stroke or transient ischaemic attack (TIA), allopurinol produced only brief, modest reductions in short-term BP variability and no effect on long-term BP variability, cerebral small vessel disease markers or cognition.^41^

These studies indicate that URAT-targeting strategies can modify renal and haemodynamic surrogates in specific contexts, but translation into clear functional or clinical CV benefits remains unproven.

## Clinical implications for cardiovascular pharmacotherapy

Taken together, epidemiology, genetics, and pharmacology support a phenotype-driven interpretation of SUA within the cardio–renal–metabolic network: hyperuricaemia is a strong risk marker and may have a modest causal role, yet outcome data do not support urate lowering as a universal strategy for cardiovascular prevention.^10,20–22,44–50^

For the purpose of this review, these phenotypes do not refer to disease-specific phenomapping models (such as HFpEF phenogroups), but rather to clinically recognizable cardio–renal–metabolic presentations in which SUA may differ in pathophysiological relevance, prognostic value, and therapeutic implications. These phenotypes are defined by the combination of symptomatic urate burden, cardiovascular context, renal/cardio–kidney–metabolic status, and metabolic profile, while sex and age are considered cross-cutting modifiers of risk thresholds and clinical expression. The main clinically relevant phenotypes are summarized in *Table 3*.

**Table 3:** Clinically relevant cardio–renal–metabolic phenotypes for the interpretation of serum uric acid and potential therapeutic implications

Clinical phenotype	Key clinical features	Interpretation of serum urate	Therapeutic relevance
Symptomatic gout/high urate-burden phenotype	Recurrent gout flares, tophi, persistent or marked hyperuricaemia, and clinically evident urate deposition.	SUA is part of an active urate-driven disease process and may contribute to systemic inflammatory burden.	Chronic urate-lowering therapy is indicated. A treat-to-target strategy remains appropriate, particularly in patients with high urate burden or recurrent flares.
Metabolic–hypertensive hyperuricaemia phenotype	Asymptomatic hyperuricaemia in the setting of obesity or central adiposity, insulin resistance or type 2 diabetes, hypertension, and early CKM involvement.	SUA mainly reflects an adverse metabolic and haemodynamic milieu, although it may also act as a risk amplifier within the cardio–renal–metabolic continuum.	Routine ULT for cardiovascular prevention is not supported. Management should focus on weight, blood pressure, glycaemic control, renal protection, and urate-friendly background therapy.
Established CAD/residual-risk phenotype	Chronic coronary artery disease or secondary prevention setting, with persistent residual risk despite guideline-directed therapy; may include patients with few standard modifiable cardiovascular risk factors.	SUA may help refine residual risk assessment, but current trial evidence does not support routine urate lowering as a cardioprotective intervention.	ULT should not be started solely for cardiovascular prevention. Priority should remain on intensive secondary prevention, with SUA considered as part of the broader risk profile.
Advanced CKD/CKM phenotype	Reduced eGFR, albuminuria, advanced CKM stage, and frequent coexistence of diabetes, hypertension, and hyperuricaemia.	SUA is closely linked to impaired renal handling and cardio–renal–metabolic burden; causal and consequential mechanisms may coexist.	Treatment should be individualized. ULT is mainly indicated for gout or symptomatic hyperuricaemia, while routine use for cardiovascular or renal protection remains unproven.
Heart failure phenotype	Heart failure with frequent diuretic exposure, renal dysfunction, congestion, and inflammatory–metabolic burden; includes both HFrEF and HFpEF contexts.	SUA often reflects disease severity, renal dysfunction, oxidative stress, congestion, and treatment background, rather than acting as an isolated therapeutic target.	In the absence of gout, routine ULT is not recommended. Management should prioritize guideline-directed heart failure therapy and reduction of avoidable urate-raising drugs when clinically feasible.

CAD, coronary artery disease; CKD, chronic kidney disease; CKM, cardio–kidney–metabolic; eGFR, estimated glomerular filtration rate; HF, heart failure; HFpEF, heart failure with preserved ejection fraction; HFrEF, heart failure with reduced ejection fraction; SUA, serum uric acid; ULT, urate-lowering therapy.

From a therapeutic perspective, current evidence supports a sex-informed rather than sex-stratified approach: women, particularly after menopause, may warrant closer attention at lower SUA levels, whereas robust sex-specific urate-lowering treatment targets for cardiovascular prevention have not been established.^6,24–26,55^ This limitation likely reflects both the observational nature of much of the sex-specific evidence and the under-representation of women in trials of urate-lowering therapies.

Within this framework, the clearest indication for chronic ULT remains gout and symptomatic hyperuricaemia. In these patients, particularly those with high cardio–renal–metabolic risk, long-term, adequately titrated XOI therapy (usually allopurinol) is appropriate, seems safe, and may provide ancillary cardiovascular benefit with sustained exposure.^42,43^ URAT1/GLUT9-directed approaches are pharmacologically attractive and genetically supported, but their role in cardiovascular care awaits definitive endpoint trials.^23,27,28^ Biologic uricases should be reserved for severe, refractory gout, even in advanced CVD, where rapid urate debulking can justify the adverse-event burden.^32–34^

For asymptomatic hyperuricaemia, current evidence does not justify routine initiation of ULT for primary or secondary cardiovascular prevention.^44–50^ Instead, SUA should be integrated into comprehensive cardio–renal–metabolic assessment and used to refine guideline-directed management. Where feasible, clinicians may preferentially select cardiometabolic drugs that are urate-neutral or urate-lowering (e.g. SGLT2 inhibitors, losartan, calcium channel blockers, statins, and fenofibrate) and limit exposure to strongly urate-raising agents (e.g. thiazides; some β-blockers) in gout-prone individuals.^32,36,39^ The urate effects of common cardiometabolic drug classes are summarized in *Table 4*.

**Table 4:** Summary of major randomized controlled trials on the cardiovascular safety and efficacy of urate-lowering therapies

Trial	Population	Comparison	Main finding	Main caveat
FAST^52^	Patients with gout and cardiovascular risk factors, already treated with allopurinol.	Febuxostat vs. allopurinol.	Febuxostat was non-inferior to allopurinol for major cardiovascular events, with no excess mortality.	Open-label design; excluded patients with severe heart failure.
CARES^56^	Patients with gout and established cardiovascular disease.	Febuxostat vs. allopurinol.	Febuxostat was non-inferior for the primary cardiovascular composite but showed higher cardiovascular and all-cause mortality.	High discontinuation and loss to follow-up rates.
ALL-HEART^44,45^	Patients with stable ischaemic heart disease but no gout.	Allopurinol plus usual care vs. usual care alone.	Allopurinol did not reduce cardiovascular death, myocardial infarction, or stroke.	Open-label design; no placebo control; substantial treatment discontinuation.

CV, cardiovascular.

Research priorities include better validation of clinically relevant phenotypes to identify patients in whom urate is a proximate driver (e.g. younger hyperuricaemia patients with early hypertension; gout with extensive atherosclerosis; advanced CKM stages) and trials that incorporate baseline urate burden, duration of exposure and achieved urate reduction.^10,23^ Drug-target MR should continue to inform target selection and dosing (e.g. SLC2A9; SLC22A11/SLC22A12), alongside interaction studies with modern cardio–renal–metabolic therapies.^23,27,57^ Finally, future studies must address persistent under-representation of women and racial/ethnic minorities in SUA-lowering trials.^55^

## Data Availability

No new data were generated or analysed in support of this research.
