# Correction to: Urate-lowering therapy in cardiovascular pharmacotherapy: from Mendelian randomization data to cardio–renal–metabolic risk modulation

**DOI:** 10.1093/ehjcvp/pvag056

**Published:** 2026-07-28

This is a correction to: Claudio Borghi, Federica Fogacci, Arrigo F G Cicero, Urate-lowering therapy in cardiovascular pharmacotherapy: from Mendelian randomization data to cardio–renal–metabolic risk modulation, *European Heart Journal - Cardiovascular Pharmacotherapy*, Volume 12, Issue 5, August 2026, Pages 410–420, https://doi.org/10.1093/ehjcvp/pvag042

The following change has been made to the originally published paper.

In the Conclusion section of the abstract, text in the last sentence is corrected to read: “[…]to identify patients with asymptomatic hyperuricemia most likely to benefit.” instead of: “[…]to identify patients most likely to benefit.”

